# Heart Rate Variability and Laboratory-Based Loss-of-Control Eating in Children and Adolescents

**DOI:** 10.3390/nu14194027

**Published:** 2022-09-28

**Authors:** Megan N. Parker, Loie M. Faulkner, Lisa M. Shank, Natasha A. Schvey, Lucy K. Loch, Hannah E. Haynes, Bess F. Bloomer, Nasreen A. Moursi, Syeda Fatima, Jennifer A. Te-Vazquez, Sheila M. Brady, Shanna B. Yang, Sara A. Turner, Marian Tanofsky-Kraff, Jack A. Yanovski

**Affiliations:** 1 Section on Growth and Obesity, Division of Intramural Research, Eunice Kennedy Shriver National Institute of Child Health and Human Development (NICHD), National Institutes of Health (NIH), 10 Center Drive, Room 1-3330, Bethesda, MD 20892, USA; 2 Department of Medical and Clinical Psychology, Uniformed Services University of the Health Sciences (USU), Bethesda, MD 20814, USA; 3 Nutrition Department, NIH Clinical Center, 10 Center Drive, Bethesda, MD 20892, USA

**Keywords:** binge-eating, overeating, heart rate, heart rate variability, child, adolescent, development, energy intake

## Abstract

Among youth, greater heart rate (HR) and lesser HR variability (HRV) are precursors to loss-of-control (LOC) eating episodes in the natural environment. However, there are limited data examining whether pre-meal HR and HRV are associated with greater LOC-eating in the laboratory setting. We therefore examined temporal relationships between pre-meal HR, frequency- and time-based metrics of pre-meal HRV, perceived LOC-eating, and energy intake during a meal designed to simulate a LOC-eating episode. Among 209 participants (54.5% female, 12.58 ± 2.72 years, 0.52 ± 1.02 BMIz), 19 reported LOC-eating in the prior month. Perceived LOC-eating during the laboratory meal was not significantly linked to pre-meal HR (*p* = 0.37), but was positively related to pre-meal HRV (ps = 0.02–0.04). This finding was driven by youth with recent LOC-eating, as these associations were not significant when analyses were run only among participants without recent reported LOC-eating (*p* = 0.15–0.99). Pre-meal HR and HRV were not significantly related to total energy intake (ps = 0.27–0.81). Additional research is required to determine whether early-stage pediatric LOC-eating is preceded by a healthy pre-meal stress response. Longitudinal studies could help clarify whether this pattern becomes less functional over time among youth who develop recurrent LOC-eating episodes.

## 1. Introduction

Pediatric loss-of-control (LOC)-eating, the subjective sense of being unable to stop eating, is associated with high weight and negative mood, e.g., [1]. Importantly, recent (within the past month) pediatric LOC-eating has been shown to predict psychological symptoms, including depressive and anxiety symptoms [2,3], excessive weight gain [4], worsened metabolic syndrome [5], and partial- and full-syndrome binge-eating disorder [2,3]. In laboratory studies, individuals with recent LOC-eating have been found to consume more energy [6], and energy from carbohydrates and highly palatable snack foods, and less energy from protein [7] compared to peers without recent reported LOC-eating. Affective theories propose that stressful psychological factors precede LOC-eating, indicating that LOC-eating may be a coping mechanism for aversive mood states, e.g., [8,9,10]. However, some [6,11,12,13], but not all [14,15], studies have failed to find significant associations between self-reported negative mood states and subsequent LOC-eating episodes. Thus, further explorations of the relationships between pre-meal affect and LOC-eating in youth are warranted.

LOC-eating appears to have physiological underpinnings. For instance, reports of recent LOC-eating have been positively associated with serum leptin [16], fasting insulin and insulin resistance [2,17,18,19], and markers of inflammation [17]. These findings are intriguing because physiological assessments are considered more objective than self-reports. However, these physiological characteristics may serve as relatively stable “trait,” rather than momentary “state”, markers of LOC-eating. Momentary state physiological markers of LOC-eating are less well understood.

Consistent with affective theories, physical markers of elevated stress may precede LOC-eating episodes. One physiological measure that is broadly considered a marker of stress [20,21,22] that may also be linked to disordered eating behavior [23], is heart rate variability (HRV; i.e., the variation between successive heart beats). Both the Polyvagal Theory of the autonomic nervous system [24,25] and the Neurovisceral Integration Model of adaptive functioning [26] hypothesize that lower HRV in response to stressors contributes to poor inhibitory control, suggesting that state changes in HRV may map onto disinhibited behavior. While stress is a broad construct and is not interchangeable with affect, it may serve as a reasonable physiological proxy. Therefore, studies utilizing HRV as an objective index of stress prior to episodes of LOC-eating may help to clarify patterns of mood states surrounding pediatric LOC-eating episodes.

Only two pediatric studies have examined heart rate (HR) and HRV in relation to LOC-eating using a temporally proximate design. Both studied adolescent girls with overweight and reported recent LOC-eating in the natural environment [27,28]. Based on multiple time points collected via ecological momentary assessment reports, results showed that HR was higher, and HRV lower, leading up to episodes of eating that were perceived as out of control. However, no study has examined HRV in relation to perceived LOC-eating and energy intake in the laboratory setting among youth.

We therefore examined if HRV immediately before a meal relates to: (1) a subjective sense of loss of control while eating and (2) objective energy intake during a meal designed to simulate a LOC-eating episode, in a large sample of males and females of a broad weight spectrum with and without recent reported LOC-eating. Consistent with affective theories of LOC-eating, e.g., [8,9,10] and with prior studies [27,28], we hypothesized that higher HR and lower HRV would be associated with greater perceived LOC-eating and energy intake. We also explored whether HRV would be related to macronutrient composition of the consumed meal. Based upon data finding that LOC-eating episodes differ from other meals in macronutrient composition [7,29], we expected that lower HRV would be linked to intake from a greater proportion of energy from carbohydrates or fats and lesser proportion of energy from protein.

## 2. Materials and Methods

### 2.1. Participants and Procedures

We conducted a secondary analysis of data collected during the initial screening visits for the NICHD Children’s Growth and Behavior Study (Clinical Trials Identifier: NCT02390765), an ongoing prospective longitudinal study investigating the relationships between eating behaviors and growth in children and adolescents. Families in the greater Washington, DC area were recruited via flyer distribution in schools, local public facilities, and mailings to surrounding communities. The study was conducted in accordance with the Declaration of Helsinki and approved by the Institutional Review Board of the National Institutes of Health. Informed consent and assent were obtained from all participants involved in the study.

Youth in good general health were eligible for the study if they were 8–17 years old, cognitively capable of understanding study procedures, and had a BMI greater than the 5th percentile for their age and biological sex. Individuals were excluded if they had a history of brain injury or major medical or psychiatric illness, current or past pregnancy, regular use of illegal drugs, recent use of medications known to affect weight, or recent weight loss. After completing a telephone screen, interested families were seen at the National Institutes of Health (NIH) Hatfield Clinical Research Center. Prior to data collection, signed informed consent and assent were obtained from parents or guardians and children, respectively. The study procedures were approved by the NIH Institutional Review Board (project identification code 15-CH-0096 and registered at NCT02390765).

### 2.2. Measures

#### 2.2.1. Demographics and Pubertal Development

Participant sex assigned at birth, age, race, and ethnicity were reported by their parent or guardian(s). Pubertal status was determined via breast development by observation and palpitation for females [30] and testicular volume by orchidometer beads and standards according to Prader for males [31]. Tanner stage I indicates no pubertal development, stages II and III indicate early- to mid-pubertal development, and stages IV and V indicate late-pubertal development. For males: pre-puberty: ≤3 mL, early- to mid-puberty: 4–12 mL, late-puberty: >12 mL.

#### 2.2.2. Anthropometric Measurements and Body Composition

Fasting body weight was measured to the nearest 0.1 kg with a calibrated scale. Height to the nearest 0.1 cm was measured in triplicate by stadiometer and averaged. Body mass index z scores were computed from height and weight according to the CDC standards for age and sex [32], and were used only to describe the sample. Percentage total body fat mass (%) and total lean body mass (kg) were determined by dual-energy X-ray absorptiometry (iDEXA system, GE Healthcare, Madison, WI, USA).

#### 2.2.3. Recent LOC-Eating

LOC-eating in the past month was assessed by the Eating Disorder Examination Interview [33,34]. For participants under 13 years of age, we utilized a version of the interview modified for children [35]; the adult version was used with older participants. These semi-structured interviews have demonstrated adequate sensitivity and specificity for detecting LOC-eating in youth and have been successfully combined in other studies, e.g., [36,37].

#### 2.2.4. Heart Rate Variability (HRV)

HRV was measured using the Zephyr BioPatch device (Zephyr Technology Inc., Baltimore, MD, USA), a non-invasive, highly reliable [38] measurement tool. Participants were seated while they wore the HRV device, with the exception of moving from one room to another or using the restroom. The HRV monitor was attached to the chest of each participant by a study coordinator approximately 30 min before the test meal and was worn until approximately 30 min after the test meal, for a total of approximately 1.5 h of wear time. This amount of time is considered sufficient to measure and compare variability among groups [39]. Pre-meal HRV was determined using time windows documented by research coordinators during the study visit; pre-meal was defined as the time period from when the device began recording data to the time the meal was served. These time windows were then entered into the Kubios HRV software (Kubios HRV Standard ver. 3.5, Kubios Oy, Kuopio, Finland) for analysis. Time and frequency domain metrics were extracted according to guidelines [40,41,42] and prior studies [27,28]. Specifically, time domain metrics were heart rate (HR), root mean square of successive RR interval (i.e., temporal distance between beats) differences (RMSSD), and percentage of successive RR intervals that differ by more than 50 ms (PNN50). The frequency domain metric was logarithmic mean of the absolute power of the high-frequency band (0.15–0.4 Hz; high frequency (HF) power).

#### 2.2.5. Buffet Test Meal

Participants were instructed to fast during the night (starting at 10:00 pm) prior to the study visit. At approximately 10:00 am, youth consumed a breakfast shake containing 21% of daily estimated energy requirements (17% protein, 16% fat, 67% carbohydrate). Between breakfast shake consumption and the buffet test meal, participants completed several tasks for the larger parent study including a comprehensive history and physical exam, the body composition DXA scan, an X-ray for skeletal age, computer-based cognitive tasks, and clinical assessment interviews. During this time participants did not engage in physical activity. At approximately 12:30 pm, participants were presented with a multi-item test meal consisting of approximately 10,000 kcal (12% protein, 32% fat, and 56% carbohydrate). They were instructed to “Let yourself go and eat as much as you want” and left alone to eat [7]. Total energy consumption (kcal) was determined using ProNutra software version 3.6.04 (Viocare, Inc., Princeton, NJ, USA) by weighing all foods and beverages. Energy intake data from some participants in this cohort have been previously reported [43,44,45,46,47].

#### 2.2.6. LOC-Eating Severity during the Test Meal

Immediately following the meal, participants self-reported rated LOC-eating severity using a series of 6 questions: “How much did you lose control during this eating episode?”, “Did you feel that you could not keep yourself from eating?”, “Did you feel that you could not stop eating once you started?”, “During the eating episode you just finished, how much did you feel a sense of loss of control?”, “How upset or distressed are you about how much you just ate?”, and “How much did you feel driven to eat?”. Participants marked their severity on a visual analog scale from 0 (“Not at all”) to 100 (“Extremely”) for each item. The arithmetic mean of these responses was used as a composite score for LOC-eating severity, as reported in prior studies [46,48]. In the current sample, Cronbach’s alpha was 0.821.

### 2.3. Statistical Analyses

Analyses were performed with IBM SPSS Statistics version 28 (SPSS, Inc., Chicago, IL, USA). To improve normality (skewness and kurtosis > −1 or < 1), we used the logarithm of HF power and of LOC-eating severity during the meal. Percentage of body fat mass and percentage of total energy intake from major macronutrients (carbohydrates, fats, and proteins) were arcsine square root transformed. Generalized linear models (GLM) were performed to investigate the associations among metrics of pre-meal HRV and eating behavior during the test meal. In separate GLMs, HRV metrics were included as independent variables and LOC-eating severity during the meal was included as the dependent variable. Four additional GLMs were run with total energy intake and percentage intake from carbohydrates, fat, and protein as dependent variables. Analyses were adjusted for sex assigned at birth (0 = female, 1 = male), race/ethnicity (0 = non-Hispanic White, 1 = other), report of LOC-eating in the prior month (0 = denied, 1 = endorsed), lean mass (kg), body fat mass (%), and age (y), as these factors have been linked to eating behavior or HRV [4,49,50,51,52]. Pubertal status was considered as a covariate due to its demonstrated effects on LOC-eating [53]; however, it was not a significant predictor in any model examining LOC-eating severity (ps = 0.9357–0.988) or total energy intake (ps = 0.263–0.376) and was therefore removed from the final presented models. After finding significant relationships, to determine if results were driven by youth with recent LOC-eating, GLMs were re-run among only those participants who did not report LOC-eating in the prior month. Given that data on the relationship between HRV and LOC-eating in youth have been in samples including only female participants [27,28], exploratory analyses were run to determine if sex moderated the relationship between HRV and eating behavior. These models revealed non-significant interactive effects of sex and HRV on LOC-eating severity (ps = 0.188–0.364) and energy intake (ps = 0.599–0.877), therefore, these interaction terms were removed before the final analyses were run.

## 3. Results

### 3.1. Participants

A total of 247 participants completed study screening visits. Among this group, 26 did not have usable HRV data, 4 were unable to complete the test meal, and 8 withdrew prior to completing study assessments. Therefore, 209 youths (84.6%) had complete data for analysis. The individuals without complete data did not differ significantly from included participants in sex, race/ethnicity, age or BMIz (ps = 0.199–0.461).

Descriptive data for included participant demographics, HRV, and test meal are provided in Table 1. Among the 209 included children and adolescents, 19 (9.1%) reported recent LOC-eating in the past month. The LOC-eating group contained 10 participants (52.6%) who reported 1 episode, 5 (26.3%) who reported 2 episodes, and 4 (21.05%) who reported 3–20 episodes in the prior month. Participants with and without recent LOC-eating did not significantly differ in age, sex, race/ethnicity, any pre-meal HRV metrics, or buffet test meal energy intake (see Table 1). Compared to participants without recent LOC-eating, those who reported recent LOC-eating had a higher percentage fat mass and reported greater LOC-eating severity during the test meal. Across all participants, average reported LOC-eating severity during the test meal was low (M = 13.94, SD = 13.56; min = 0.00, max = 67.50), but perceived severity was positively associated with energy intake during the meal (F (1, 202) = 4.77, *p* = 0.030, ß = 66.768). LOC-eating severity during the test meal was not significantly related to percentage intake from carbohydrate, fat or protein (ps > 0.520).

### 3.2. Pre-Meal HRV, LOC-Eating Severity, and Energy Intake

**Figure 1:**
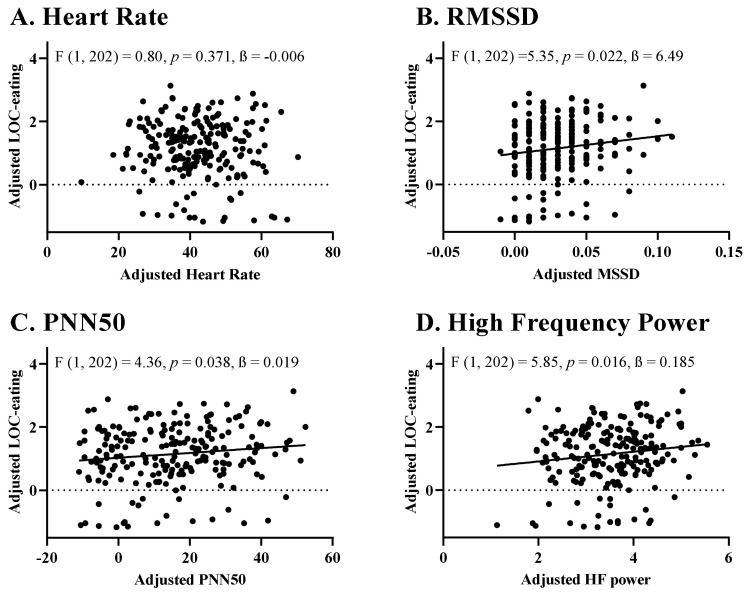
Associations of Pre-Meal Heart Rate Variability Metrics and Perceived LOC-Eating During a Laboratory Buffet Meal. (**A**) Pre-Meal Heart Rate and Perceptions of LOC-eating; (**B**) Pre-Meal RMSSD and Perceptions of LOC-eating; (**C**) Pre-Meal PNN50 and Perceptions of LOC-eating; (**D**) Pre-Meal High Frequency Power and Perceptions of LOC-eating. LOC-eating, loss-of-control eating in the past month; RMSSD, Root mean square of successive RR interval differences; PNN50, Percentage of successive RR intervals that differ by more than 50 ms; Log HF Power, logarithmic mean of the absolute power of the high-frequency band (0.15–0.4 Hz). Models were adjusted for sex assigned at birth (0 = Female, 1 = Male), race/ethnicity (0 = Non-Hispanic White, 1 = Other), report of LOC-eating in the prior month (0 = denied, 1 = endorsed), lean mass (kg), percentage body fat mass, and age.

Across the entire sample, greater pre-meal HR was not significantly associated with LOC-eating severity (F (1, 202) = 0.80, *p* = 0.371, ß = −0.006); Figure 1A). In contrast, HRV as measured by RMSSD (F (1, 202) = 5.35, *p* = 0.022, ß = 6.490), PNN50 (F (1, 202) = 4.36, *p* = 0.038, ß = 0.019) and HF Power (F (1, 202) = 5.85, *p* = 0.016, ß = 0.185) were positively linked to LOC-eating severity during the meal such that higher HRV preceded greater perceived LOC-eating (Figure 1B–D). No metrics of HRV were linked to total energy intake (ps = 0.274–0.813). RMSSD was positively linked to percentage energy intake from fat (F (1, 202) = 4.604, *p* = 0.033, ß = 0.497), but no other HRV metric was significantly linked to percentage intake of energy from carbohydrate, fat or protein during the meal (ps = 0.073–0.631).

When the analyzed data were restricted only to participants who did not report recent LOC-eating, none of the HRV metrics were significantly associated with LOC-eating severity (ps = 0.148–0.989), total energy intake (ps = 0.270–627), or percentage intake from carbohydrate or protein (ps = 0.070–0.740). Percentage intake from fat was positively associated with RMSSD (*p* = 0.028), but not with other metrics of HRV (ps = 0.065–0.083).

## 4. Discussion

In this study of generally healthy males and females of a broad age and weight strata, we examined whether pre-meal HR and HRV were associated with the perception of LOC-eating and energy intake during a laboratory-based test meal. In contrast to expectations, higher pre-meal HRV was associated with perceptions of greater LOC-eating, which was driven primarily by participants who had reported LOC-eating in the past month. No significant relationships emerged between any autonomic indices and energy intake during the meal.

The lack of association between HR and LOC-eating and the positive association between HRV and LOC-eating perception are in contradistinction with our hypotheses. We found a positive association between pre-meal HRV and LOC-eating among all participants; however, among those who did not report recent LOC-eating, there was no association between HRV and perceived LOC-eating. Thus, the presentation of a large buffet of foods may possibly elicit physiological markers of the stress response in youth with LOC-eating. Youth who do not engage in LOC-eating do not appear to evince a link between HR/HRV prior to a meal and eating behavior. Although past studies have observed a maladaptive stress response in adolescents with frequent LOC-eating prior to an episode of LOC-eating [27,28], our findings appear indicative of a healthful pre-meal stress response among youth with infrequent recent LOC-eating. This pattern of pre-meal stress responses may help to explain why prior studies have failed to detect a link among self-reported pre-meal negative affective states and LOC-eating in youth [6,11,12,13,54], despite consistent evidence for this relationship in adults with eating disorders [55,56,57]. Additionally, our findings are consistent with affective models of LOC-eating that are predicated on the notion that eating is used as a method to manage stressful circumstances, e.g., [8,9,10], thus potentially rendering a healthy stress response at early stages of disordered eating onset. We posit that the stress response becomes impaired only once a repeated pattern of using food to cope with aversive states has developed.

Consistent with this notion, our sample reported less frequent LOC-eating compared to the samples in the Ranzenhofer et al. studies [27,28], which served as the basis for our hypotheses. In both of these studies, only adolescents with recent LOC-eating at a threshold of at least two episodes in the prior month were studied. In our sample, approximately 50% of participants with recent LOC-eating reported only 1 episode of LOC-eating in the prior month. Likewise, weight may play a role in a dysfunctional pre-meal HR/HRV response. The mean BMIz and BMI percentile of our participants with recent LOC-eating were 0.88 and 62.6, respectively. Conversely, participants in the Ranzenhofer studies had a mean BMIz of 2.17 in one study [27] (indicating most participants had severe obesity) and a mean BMI percentile of 92.6 in the other [28] (suggesting most participants had overweight or obesity). Another notable difference is our inclusion of males. Although we did not observe different sex differences in the relationship between HRV metrics and eating behavior, sex differences in the frequency of LOC-eating behaviors emerge in late adolescence [58,59,60]. Thus, future studies should probe for potential sex-based differences in the link between autonomic function and disordered eating. Examining pre-meal HR/HRV in relation to the development of recurrent LOC-eating and weight gain over time in males and females may help to identify key factors in the transition from healthy to dysfunctional physiological stress responses to food, as well as exacerbated disordered eating among youth.

We cannot rule out the contribution of methodological differences to our opposing findings. Given that our meal was conducted in a controlled laboratory environment, we may not have captured individual fluctuations in stress that may precede eating episodes in the natural environment. We also studied only one episode of eating; assessment at multiple timepoints may reveal different patterns. Outside of between-study differences in samples and methodologies, individual differences in factors known to impact both HR/HRV and eating behavior, such as sleep [61,62] and physical activity [63,64], may also complicate the association between pre-meal autonomic function and eating behavior in youth. Additionally, in the present study, participants were not systematically told exactly when they would eat lunch, though they knew a lunch meal would be provided. Anticipation of events that are stressful has been shown to impact an individual’s physiological activity prior to and following a stressful event [65]. This phenomenon has not been studied within the context of eating behavior. Yet, it is possible that youth with LOC-eating could view a meal as stressful, and anticipation of eating could alter their autonomic function. Thus, the potential impact of anticipation should be considered in future studies.

In contrast to expectations, pre-meal HRV was not significantly related to either total or specific macronutrient intake at the meal. This finding may again reflect our sample characteristics and study methodology. Unlike studies in the natural environment with multiple mealtime assessments, a single test meal may not effectively capture enough data points to detect associations between HR/HRV and intake. Alternatively, among youth with LOC-eating, autonomic functioning before a meal may be more important to the perception of loss-of-control compared with objective intake during the meal.

Perceived LOC-eating during the laboratory meal was significantly and positively related to total energy intake among our sample and was higher among those with recent LOC-eating compared to participants who reported no recent LOC-eating. This suggests that self-reported LOC-eating severity during a laboratory meal may be a useful measure of this disordered eating behavior in youth outside the laboratory environment. With regard to energy intake, extant data are mixed on the link between reported LOC-eating and total energy consumed in laboratory studies, with some, e.g., [6], but not all, e.g., [7], samples showing an association. Prior pediatric studies with a laboratory meal have compared intake between youth with and without recent LOC-eating (e.g., in the prior 1–3 months); our findings are consistent with a prior study that tested for an association between children’s total energy intake and their perceptions of LOC-eating during a laboratory meal [6]. In our sample, perceived LOC-eating during the meal was not related to percentage intake of any macronutrient. Additionally, the finding that no relationship was identified with recent LOC-eating and meal macronutrient composition is perplexing and these associations warrant additional exploration. Although this measure of perceived LOC-eating has demonstrated initial concordance with energy intake and recent LOC-eating, the extent to which perceived LOC-eating in the laboratory correlates with perceived LOC-eating in the natural environment, and whether it is predictive of future disordered eating behavior in youth, has yet to be determined.

Strengths of this study include the use of well-validated measurement tools such as the Zephyr BioPatch, DEXA, and clinical interviews to measure recent LOC-eating. We also studied both time- and frequency-domain metrics of HRV. The sample was relatively large and racially and ethnically diverse. Study limitations include the use of a self-reported LOC-eating severity measurement tool based on an average of the response to 6 questions; however, the questions were modeled on those used to assess for LOC-eating episodes in the EDE structured interview. Additionally, a relatively small number of participants reported recent LOC-eating which prevented us from testing whether the associations between pre-meal HRV and perceived LOC-eating vary by frequency of recent LOC-eating. The use of a laboratory test meal might be considered a strength because intake could be carefully measured, but such in-lab buffet meals may not be ecologically valid. Repeated measurement of HRV and eating behavior in more naturalistic environments may help to elucidate whether the predictions from affective models contribute to dysregulated eating behaviors in youth.

## 5. Conclusions

Overall, our study results provide important considerations for studying affective models of disordered eating among generally healthy children and adolescents. Youth who may be in the early stages of disordered eating behavior development appear to display an adaptive stress response prior to meals. This is somewhat consistent with affective theories; however, when and why—a healthy stress response becomes a maladaptive stress response in youth with LOC-eating, and whether this transition maps onto changes in disordered eating symptomatology, remain unclear. Examination of other biomarkers of stress, such as cortisol, or appetite, for example leptin and ghrelin, may provide additional insight into momentary physiological correlates of LOC-eating. Additionally, longitudinal and multimethod studies could help to identify the factors that promote a transition from healthy to maladaptive pre-meal stress responses in youth with LOC-eating.

## Figures and Tables

**Table 1:** Participant characteristics and descriptive data.

Variable	Total Sample (N = 209)	LOC-Eating (*n* = 19)	No LOC-Eating (*n* = 190)		
	*n*, %	*n*, %	*n*, %	X^2^	*p*
Sex (% Female) ^1^	114, 54.5	14, 73.7	100, 52.6	3.09	0.079
Ethnicity/Race ^2^				1.52	0.218
Non-Hispanic Asian	25, 12.0	24, 12.6	1, 5.3		
Non-Hispanic Black	56, 26.8	50, 26.3	6, 31.6		
Non-Hispanic Multiracial	17, 8.10	16, 8.4	1, 5.3		
Non-Hispanic White	94, 45.0	88, 46.3	6, 31.6		
Non-Hispanic Unknown	1, 0.5	1, 0.5	0, 0.0		
Hispanic Black	2, 1.0	2, 1.1	0, 0.0		
Hispanic Multiracial	5, 2.4	4, 2.1	1, 5.3		
Hispanic White	8, 3.8	4, 2.1	4, 21.1		
Hispanic Unknown	1, 0.5	1, 0.5	0, 0.0		
Pubertal stage ^3^				1.75	0.416
Pre-pubertal	46, 22.0	4, 21.1	42, 22.6		
Mid-pubertal	44, 21.1	2, 10.5	42, 22.6		
Late pubertal	115, 55.0	13, 68.4	102, 54.8		
	M, SD	M, SD	M, SD	*t*	*p*
Age (years)	12.6, 2.7	13.7, 2.7	12.5, 2.7	−1.86	0.064
BMIz	0.52, 1.02	0.88, 1.02	0.49, 1.02	−1.60	0.111
Percent Fat Mass (%)	0.55, 0.10	0.61, 0.09	0.55, 0.10	−2.43	0.016
Mean HR (beats/min)	83.0, 11.8	84.0, 12.0	83.0, 10.9	0.34	0.735
RMSSD (ms)	0.059, 0.02	0.06, 0.02	0.05, 0.02	1.27	0.205
PNN50 (%)	30.2, 15.8	30.6, 15.6	26.9, 18.2	0.95	0.344
Log HF Power (ms ^2^)	7.0, 0.9	7.1, 0.8	6.7, 1.0	1.67	0.096
LOC-Eating Severity ^4^	13.9, 13.6	31.8, 18.6	12.2, 11.6	−4.50	<0.001
	M, SD	EMM, SE	EMM, SE	F	*p*
Energy Intake (kcal) ^5^	980.3, 419.8	945.0, 95.0	991.0, 29.4	0.21	0.645

Note. LOC-eating, loss-of-control eating in the past month; X^2^, chi-square; BMIz, body mass index (kg/m^2^) standardized z-score [30]; HR, heart rate; RMSSD, Root mean square of successive RR interval differences; PNN50, Percentage of successive RR intervals that differ by more than 50 ms; Log HF Power, logarithmic mean of the absolute power of the high-frequency band (0.15–0.4 Hz); EMM, estimated marginal mean, SE, standard error of the mean. No LOC-eating was set as the reference group in *t*-tests. ^1^ Sex only assessed Male and Female sex assigned at birth. ^2^ A chi-square test compared non-Hispanic white vs. other. ^3^ Four participants did not undergo Tanner staging. Percentages were out of a total 205 youth for the total sample, and 186 youth for the No LOC-eating group. ^4^ Equal variances not assumed. ^5^ The generalized linear mixed model comparing energy intake by LOC-eating group was adjusted for age, sex, race/ethnicity, and percent body fat mass.

## Data Availability

Data available on request.
